# Anemia as a Significant Predictor of Adverse Outcomes in Hospitalized Patients With Acute Exacerbations of Chronic Obstructive Pulmonary Disease: Analysis of National (Nationwide) Inpatient Sample Database

**DOI:** 10.7759/cureus.34343

**Published:** 2023-01-29

**Authors:** Hardik Sonani, Kartik Dhaduk, Nilesh Dankhara, Krina Viroliya, Chintan Desai, Ashish A Sonani, Aditya S Patel, Venkataraman Palabindala, Ashish M Goti, Jagdish Desai

**Affiliations:** 1 Department of Pathology, University of Mississippi Medical Center, Jackson, USA; 2 Department of Internal Medicine, Geisinger Health System, Wilkes-Barre, USA; 3 Department of Pediatrics, University of Mississippi Medical Center, Jackson, USA; 4 Department of Internal Medicine, HCA Houston Healthcare Kingwood/University of Houston, Kingwood, USA; 5 Office of Oral Health, Mississippi State Department of Health, Jackson, USA; 6 Department of Pulmonary Medicine, New Civil Hospital, Surat, Surat, IND; 7 Department of Public Health, Jackson State University, Jackson, USA; 8 Department of Internal Medicine, University of Mississippi Medical Center, Jackson, USA; 9 Department of Pediatrics, Ochsner Medical Center, New Orleans, USA; 10 Department of Pediatrics, Tulane University School of Medicine, New Orleans, USA; 11 Deapartments of Pediatrics and Child Health, Neonatology, Pediatric Intensive Care, Nice Children Hospital, Surat, IND; 12 Department of Pediatrics/Neonatology, Pediatrix Medical Group, Austin, USA

**Keywords:** acute exacerbation copd, anemia, copd, hemoglobin, mortality, resource utilization, respiratory illness, ventilation

## Abstract

Background: Acute exacerbation of chronic obstructive pulmonary disease (AECOPD) has significant health implications. Anemia is usually an unseen comorbidity, which could significantly affect outcomes in AECOPD patients, and there is limited data to support this. We conducted this study to assess the effect of anemia on this patient population.

Methods: We performed a retrospective cohort study using the National (Nationwide) Inpatient Sample (NIS) data from 2008 to 2014. Patients with AECOPD and anemia with age >40 years were identified using appropriate International Classification of Diseases, Ninth Revision (ICD-9) codes, excluding transfer out to other hospitals. We calculated the Charlson Comorbidity Index as a measure of associated comorbidities. We analyzed bivariate group comparisons in patients with and without anemia. Odds ratios were calculated using multivariate logistic and linear regression analysis using SAS version 9.4 (2013; SAS Institute Inc. Cary, North Carolina, United States).

Results: Among 3,331,305 patients hospitalized with AECOPD, 567,982 (17.0%) had anemia as a comorbidity. The majority of patients were elderly, women, and white. After adjusting for potential confounders in regression, mortality (adjusted OR (aOR) 1.25, 95%CI: 1.18-1.32), length of hospital stay (β 0.79, 95%Cl 0.76-0.82), and hospitalization cost (β 6873, 95%Cl 6437-7308) were significantly higher in patients with anemia. In addition, patients with anemia required significantly higher blood transfusion (aOR 16.9, 95%CI 16.1-17.8), invasive ventilator support (aOR 1.72, 95%CI 1.64-1.79), and non-invasive ventilator support (aOR 1.21, 95%CI 1.17-1.26).

Conclusion: In this first retrospective largest cohort study on this topic, we find anemia is a significant comorbidity associated with adverse outcomes and healthcare burden in hospitalized AECOPD patients. We should focus on close monitoring and management of anemia to improve the outcomes in this population.

## Introduction

Chronic Obstructive Pulmonary Disease (COPD) is a disease of the lung characterized by chronic obstruction of airflow that interferes with normal breathing [1]. COPD is very prevalent with nearly 12 million diagnosed cases in the United States and it is the third leading cause of death [2], placing a substantial burden on our healthcare system that was estimated to be 32 billion dollars in 2010 [3]. In addition to causing high healthcare costs, COPD imposes a significant burden in terms of disability, impaired quality of life, and limitation in work capacity that is related to the substantial indirect cost to the healthcare system [3]. It is not reversible but is one of the common controllable diseases. The progressive reduction of the airflow from enhancing chronic inflammatory changes in the lungs with infection or exposure to environmental irritants can result in acute exacerbation of COPD (AECOPD) [1]. Comorbidities are common and important risk factors in the quality of life of these patients [4]. The most common comorbidities associated with AECOPD include ischemic heart disease, heart failure, diabetes mellitus, hypertension, anemia, osteoporosis, depression, and anxiety [5,6]. Early detection and the best possible management of the comorbidities may reduce adverse outcomes [7] and healthcare-related burdens in these patients.

In recent years, anemia has gained importance in patients with COPD [8]. According to the World Health Organization (WHO), anemia is defined as a hemoglobin (Hb) level of less than 13 g/dL in males and 12 g/dL in females [9]; however, it is debatable to what extent anemia is associated with adverse outcomes in critically ill COPD patients requiring invasive or non-invasive modes of ventilation and how it should be managed. The mechanism of anemia in COPD is complex but anemia of chronic disease is probably the predominant mechanism [8]. It is difficult to measure the direct impact of anemia in these patients due to the presence of multiple comorbidities but its association and impact on quality of life and mortality are well documented in many other chronic diseases such as chronic kidney disease, malignancy, congestive heart failure, inflammatory bowel disease, etc. [10-13]. Although studies have shown that anemia can lead to adverse outcomes in COPD [8], there is a paucity of recent epidemiologic trends in AECOPD hospitalization. To the best of our knowledge, there is no current large-scale population-based study available evaluating the impact of anemia on mortality with controlling the effects of confounders.

In our study, we hypothesized that anemia continues to adversely affect the outcomes in patients admitted with AECOPD. We aimed to estimate the true impact of anemia on inpatient mortality, length of hospital stay (LOS), resource utilization, and hospitalization cost in a cohort of AECOPD patients.

## Materials and methods

Data source

Healthcare Cost and Utilization Project (HCUP) is the largest collection of longitudinal hospital care data in the United States, funded by the Agency for Healthcare Research and Quality (AHRQ) [14]. We studied a retrospective cohort of patients with AECOPD using the National (Nationwide) Inpatient Sample (NIS) database sponsored by HCUP entries from 2008 to 2014 (seven years span). Our study population included patients admitted with chronic obstructive bronchitis with acute exacerbation (International Classification of Diseases, Ninth Revision (ICD-9) code of 491.21) as a primary discharge diagnosis [15]. Then, patients with anemia identified consisted of clinical classification software (CCS) diagnosis codes (59 and 61) in any of the discharge diagnosis fields (DX2-DX25). Patients who were younger than 40 years, transferred out to other hospitals, and missing information (age, gender, and inpatient mortality) were excluded from the analysis. To identify resource utilization among the patient population, procedure codes for non-invasive positive pressure ventilation (NIPPV) (93.90), invasive mechanical ventilation (IMV) (967.0, 967.1, and 967.2), and blood transfusion (99.00, 99.01, 99.02, 99.03, and 99.04) were used. Prior studies have shown that ICD-9, Clinical Modification (ICD-9-CM) codes for MV [16] and NIV [17] (sensitivity 78%; specificity 100%) are reliable. 

Variables

Key outcomes were in-hospital mortality, LOS, resource utilization (non-invasive or invasive ventilation, blood transfusion), and cost of hospitalization. We recorded demographic variables, including patients’ age, gender, race (White, Black, Hispanic, or other), primary expected payer (Medicare, Medicaid, Private, or other), and median household income. We also recorded hospital-level variables, including a region (Northeast, Midwest, South, and West), control (Government or Private), location (Rural or Urban), and bed-size (Small, Medium, or Large) for the selected patient population.

Statistical analysis

We performed weighted statistical analysis using a complex survey design of the NIS to represent national estimates. Bivariate group comparisons were made. We used the survey-weighted Chi-square test for categorical data and the t-test for continuous data. Z-tests for proportions were used for multi-categorical variables in the bivariate analyses, with the p-value adjusted with Bonferroni correction for multiple comparisons. We had done a multivariate logistic regression analysis to obtain adjusted odds ratios (aOR) by accounting for hospital-level and patient-level variation in outcomes simultaneously. We specifically compared inpatient mortality, LOS, total hospital charges, and resource utilization between patients with and without anemia. We used the Cochrane-Armitage test for linear trends over the study period from 2008 to 2014. All statistical tests were performed with a significance level set at a p-value of 0.01. Odds ratio and β-value were reported with 95% confidence intervals (CIs) throughout our analysis. We calculated the Charlson Comorbidity Index (CCI) as a measure of associated comorbidities. All analyses were performed with SAS version 9.4 (2013; SAS Institute Inc. Cary, North Carolina, United States).

## Results

There were 3,401,897 (1.32%) patients admitted with AECOPD between 2008 and 2014. After the exclusion of patients younger than 40 years (N=21416) and transfer to other hospitals (N=49176), there were 3,331,305 admissions available for analysis. Among them, 567,982 (17.04%) had anemia as a comorbidity. The majority of patients were more than 70 years old (58.6%), and more women admitted than men (57.4% vs. 42.6%) were. White had a lower prevalence compared to Black (16.5% vs. 20.9%). Overall, the patient population with anemia consisted of White (71.0%), Black (11.7%), Hispanic (4.2%), and others (3.1%). Most of the patients had Medicare insurance status (79.7%) and less than $39,000 median household income (34.8%). Among hospital-level variables, the majority of the patients were from the south region (42.7%), urban-nonteaching hospitals (43.7%), and large bed-size hospitals (55.4%) (Table 1). Patients admitted for AECOPD with anemia had a higher number of diagnoses (14 ± 0.05 vs. 10 ± 0.04; P<.001) and a higher level of comorbid illness (CCI, 2.8 vs 2.0; P<.001). In addition, patients with anemia required longer hospitalizations of four days (interquartile range (IQR), 2-5) vs three days (IQR, 3-6); P<.001, and acquired higher charges ($33,587 ± 141 vs $24,075 ± 42; P<.001) than those who did not have anemia at discharge. 

**Table 1:** Baseline characteristics NIPPV: non-invasive positive pressure ventilation; IMV: invasive mechanical ventilation; BT: blood transfusion; CCI: Charlson Comorbidity Index. *P-value adjusted with Bonferroni correction.

Characteristics	No Anemia (%) or mean ± SE	Anemia N (%) or mean ± SE	Total N (%) or mean ± SE	P-value
All patients	2,763,323 (82.95)	567,982 (17.05)	3,331,305 (100)	-
Age (years) *				
40-50	185,497 (6.7)	18,757 (3.3)	204,254 (6.1)	<0.001
51-60	559,685 (20.3)	70,990 (12.5)	630,675 (18.9)	<0.001
61-70	796,677 (28.8)	145,371 (25.6)	942,048 (28.3)	<0.001
>70	1,221,464 (44.2)	332,864 (58.6)	1,554,328 (46.7)	<0.001
Gender				
Male	1,241,752 (44.9)	241,694 (42.6)	1,483,446 (44.5)	<0.001
Female	1,521,571 (55.1)	326,288 (57.4)	1,847,859 (55.5)	<0.001
Race*				
White	2,025,364 (73.3)	403,052 (71.0)	2,428,416 (72.9)	<0.001
Black	251,398 (9.1)	66,474 (11.7)	317,872 (9.5)	<0.001
Hispanic	105,962 (3.8)	23,942 (4.2)	129,904 (3.9)	<0.001
Others	84,981 (3.1)	17,942 (3.1)	102,923 (3.1)	<0.001
Missing	295,618 (10.7)	56,572 (10.0)	352,190 (10.6)	<0.001
Primary Expected Payer (uniform) *				
Medicare	1,930,605 (69.9)	452,862 (79.7)	2,383,467 (71.5)	<0.001
Medicaid	305,451 (11.0)	46,427 (8.2)	351,878 (10.6)	<0.001
Private	356,116 (12.9)	49,805 (8.8)	405,921 (12.2)	<0.001
Others	165,348 (6.0)	17,922 (3.1)	183,270 (5.5)	<0.001
Missing	5803 (0.2)	966 (0.2)	6769 (0.2)	<0.001
Median Household Income*				
$1 - $38,999	981,684 (35.5)	197,799 (34.8)	1,179,483 (35.4)	<0.001
$39,000 - $47,999	783,925 (28.4)	153,857 (27.1)	937,782 (28.2)	<0.001
$48,000 - $62,999	558,448 (20.2)	120,232 (21.2)	678,680 (20.4)	<0.001
$63,000 or more	373,154 (13.5)	84,037 (14.8)	457,191 (13.7)	<0.001
Missing	66,112 (2.4)	12,057 (2.1)	78,169 (2.3)	<0.001
Hospital Region*				
Northeast	523,770 (19.0)	101,264 (17.8)	625,034 (18.8)	<0.001
Midwest	668,059 (24.2)	141,887 (25.0)	809,946 (24.3)	<0.001
South	1,212,920 (43.9)	242,753 (42.7)	1,455,673 (43.7)	<0.001
west	358,574 (12.9)	82,078 (14.5)	440,652 (13.2)	<0.001
Teaching Location*				
Rural	641,446 (23.2)	111,686 (19.7)	753,132 (22.6)	<0.001
Urban nonteaching	1,176,186 (42.5)	248,292 (43.7)	1,424,478 (42.8)	<0.001
Urban teaching	929,791 (33.7)	204,426 (36.0)	1,134,217 (34.0)	<0.001
Missing	15,900 (0.6)	3,578 (0.6)	19,478 (0.6)	<0.001
Hospital Volume*				
Small	524,417 (19.0)	96,125 (17.0)	620,542 (18.6)	<0.001
Medium	738,497 (26.7)	153,169 (27.0)	891,666 (26.8)	<0.001
Large	1,484,508 (53.7)	315,111 (55.4)	1,799,619 (54.0)	<0.001
Missing	15,901 (0.6)	3,577 (0.6)	19,478 (0.6)	<0.001
Resource Utilization				
NIPPV	140,237 (5.1)	37,123 (6.5)	177,360 (5.3)	<0.001
IMV	52,622 (1.9)	19,885 (3.5)	72,507 (2.2)	<0.001
BT	18,395 (0.7)	62126 (10.9)	80521 (2.4)	<0.001
Number of diagnoses	10 ± 0.04	14 ± 0.05	11 ± 0.006	<0.001
CCI	2.0 ± 0.001	2.8 ± 0.005	2.2 ± 0.001	<0.001

We constructed a multivariable regression model for predicting inpatient mortality, in order to evaluate the independent main effect of anemia on mortality. The mortality risk is higher in old age (>70 years old) (aOR=4.07; p<0.001) and lesser in females (aOR=0.79; p<0.001), Blacks (aOR=0.60; p<0.001) compared to males and Whites, respectively. The most significant predictors of mortality among AECOPD patients with anemia were utilization of resources like blood transfusion (aOR=1.67; p<0.001), NIPPV (aOR=2.84; p<0.001) and IMV (aOR=21.6; p<0.001). Death among patients with >10 CCI was also significantly higher (aOR=3.41; p<0.001) (Table 2).

**Table 2:** Predictors of Inpatient Mortality in AECOPD patients with anemia The model used: Mixed-effects Survey-Weighted Logistic Regression. Additionally adjusted by (not shown in table): Primary Payer, Median Household Income, Hospital Bed Size, Hospital Control, and Urban location of the hospital. AECOPD: acute exacerbation of chronic obstructive pulmonary disease

Variables	Adjusted Odds Ratio	Lower 95% value	Upper 95% value	P- value
Age (years)				
40-50	Reference			
51-60	1.56	0.98	2.48	0.061
61-70	2.06	1.29	3.29	0.002
>70	4.07	2.54	6.50	<0.0001
Female (male=reference)	0.79	0.72	0.87	<0.0001
Race				
White	Reference			
Black	0.60	0.51	0.70	<0.0001
Hispanic	0.80	0.64	1.01	0.06
Others	1.05	0.84	1.32	0.62
Procedures				
BT	1.67	1.49	1.87	<0.0001
NIPPV	2.84	2.49	3.24	<0.0001
IMV	21.6	19.4	24.1	<0.0001
Charlson Comorbidity Index				
<2	Reference			
2-4	1.09	0.97	1.22	0.13
5-7	1.22	1.04	1.42	0.01
8-10	2.66	2.05	3.45	<0.0001
>10	3.41	1.68	6.93	0.0007

After adjusting for potential confounders in regression, in-hospital mortality (aOR 1.25, 95%CI 1.18-1.32), LOS (β 0.79, 95%Cl 0.76-0.82) and hospitalization cost (β 6873, 95%Cl 6437-7308) were significantly higher in patients with anemia in compared to patients without anemia. In addition, patients with anemia required significantly higher invasive ventilator support (aOR 1.72, 95%CI 1.64-1.79) and non-invasive ventilator support (aOR 1.21, 95%CI 1.17-1.26), as well as a higher need for blood transfusion (aOR 16.9, 95%CI 16.1-17.8) (Table 3).

**Table 3:** Multivariate regression analysis for outcomes in AECOPD patients with and without anemia *Multivariate logistic regression;  ^#^Multivariate linear regression. Controlled for age, sex, race, socioeconomic status, insurance type, Charlson Comorbidity Index, and hospital characteristics NIPPV: noninvasive positive pressure ventilation; IMV: mechanical ventilation; BT: blood transfusion; LOS: length of stay; IQR: interquartile range; SEM: standard error of mean; AECOPD: acute exacerbation of chronic obstructive pulmonary disease

Characteristics	No Anemia N (%)	Anemia N (%)	Unadjusted OR (95% CI)* or p-value	Adjusted OR (95% CI)* or β-value (95%Cl)^#^
Mortality, %	35,589 (1.2) ± 725 (0.01)	11,817 (2.0) ± 322 (0.04)	1.62 (1.55-1.71)	1.25 (1.18-1.32)
Need of NIPPV	140,237 (5.1) ± 3208 (0.09)	37,123 (6.5) ± 1178 (0.17)	1.30 (1.26-1.35)	1.21 (1.17-1.26)
Need of IMV	52,622 (1.9) ± 979 (0.02)	19,885 (3.5) ± 481 (0.06)	1.86 (1.79-1.94)	1.72 (1.64-1.79)
Need of BT	18395 (0.6) ± 512 (0.01)	62126 (10.9) ± 1237 (0.15)	18.3 (17.5-19.1)	16.9 (16.1-17.8)
LOS in survivors, days (Median, IQR)	3.0 (3.0)	4.0 (3.0)	<0.0001	0.79 (0.76-0.82)
Total charges, $ (Mean, SEM)	24,075 ± 42	33,587 ± 141	<0.0001	6873 (6437-7308)

**Figure 1:**
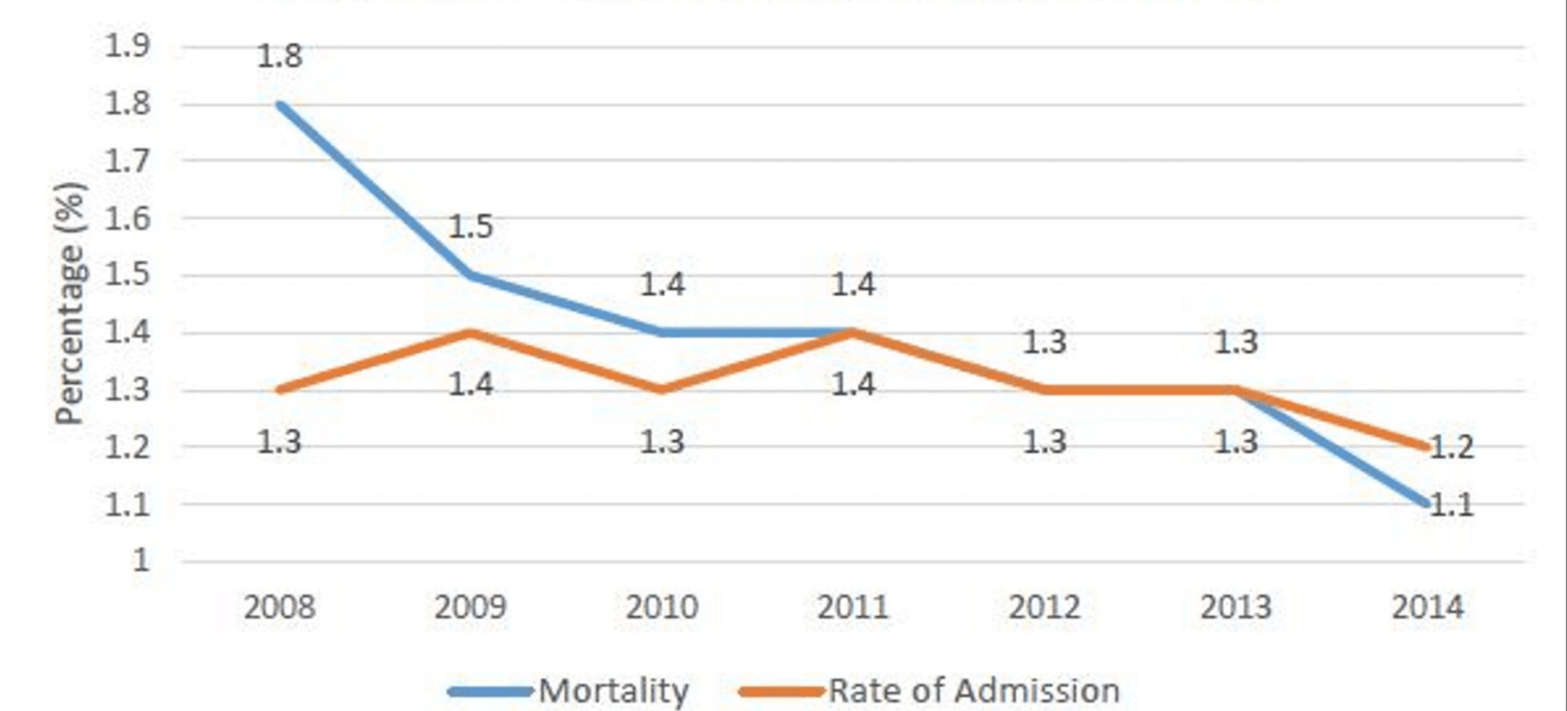
Rate of admission and mortality for AECOPD AECOPD: acute exacerbation of chronic obstructive pulmonary disease

**Figure 2:**
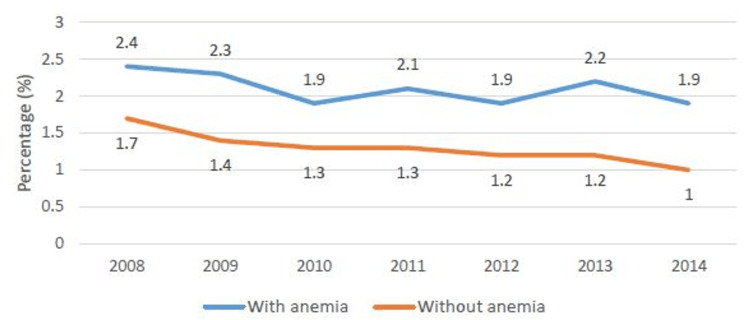
Mortality rate in AECOPD patients with and without anemia AECOPD: acute exacerbation of chronic obstructive pulmonary disease

The rate of AECOPD admissions remained stable (from 1.3% in 2008 to 1.2% in 2014) whereas the rate of mortality decreased over time (1.8% in 2008 to 1.1% in 2014) (Figure 1). However, the mortality rate remained high in anemic patients compared to non-anemic patients (2.4% vs 1.7% in 2008 and 1.9% vs 1% in 2014) (Figure 2).

**Figure 3:**
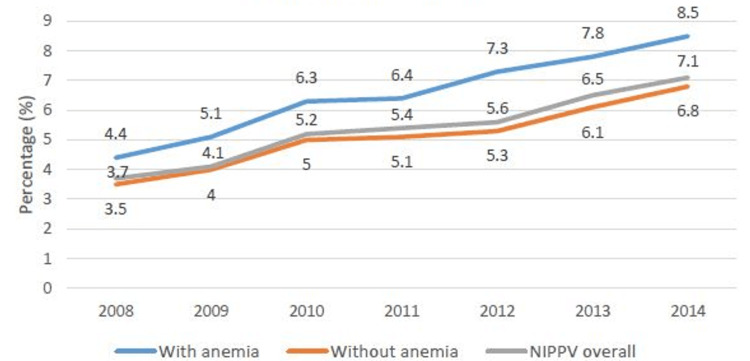
Utilization of NIPPV in AECOPD patients with and without anemia NIPPV: noninvasive positive pressure ventilation; AECOPD: acute exacerbation of chronic obstructive pulmonary disease

**Figure 4:**
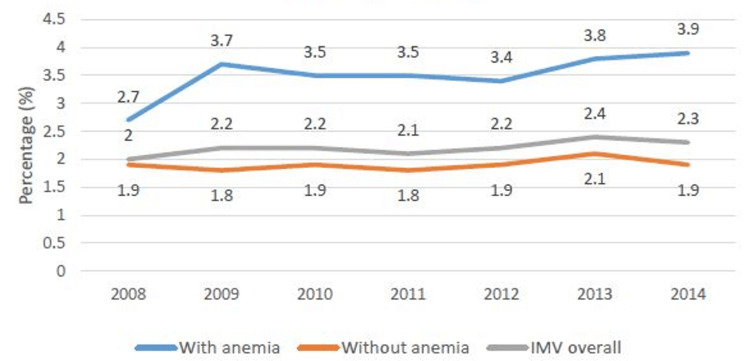
Utilization of invasive ventilation in AECOPD patients with and without anemia. AECOPD: acute exacerbation of chronic obstructive pulmonary disease; IMV: invasive mechanical ventilation

We also found a higher utilization rate of NIPPV compared to IMV. As depicted in Figure 3 and Figure 4, the presence of anemia is associated with higher use of NIPPV (4.4% vs 3.5% in 2008 and 8.5% vs 6.8% in 2014) as well as IMV (2.7% vs 1.9% in 2008 and 3.9% vs 1.9% in 2014) compared to non-anemic patients. We find an upward trend in the use of NIPPV (3.7% in 2008 to 7.1% in 2014) in AECOPD management but the trend of IMV use remained stable (2% in 2008 to 2.3% in 2014) over the study period, suggesting changing trend of ventilation use in high-risk patients.

## Discussion

COPD is associated with multiple comorbidities and anemia is one of the important ones that could be easily missed in the setting of acute exacerbation and complex hospital course. The prevalence of anemia was found to be 17% in the current study, which is consistent with the reported overall anemia prevalence in COPD [8,18]. Anemia is an easily detectable and modifiable factor that is associated with significant resource utilization, healthcare-related cost, mortality, and LOS as described in this study in AECOPD patients. 

The pathophysiology of anemia development includes multiple mechanisms. The association of anemia in COPD and its effect on respiratory physiology is reported in a few studies. There is no clear cut-off value or guideline for the threshold value of the Hb in COPD for better outcomes, but few studies have thrown some light on this topic. Chambellan et al. reported each 5% increase in the hematocrit is associated with improved survival and hematocrit was the strongest prognostic factor next to age [19]. Kollert published optimal cut-off values of 14.3 g/dl for females and 15.1 g/dl for males for long-term survival prediction in AECOPD [18]. Interestingly, two studies have shown a Hb value of more than 12 gm/dl for better outcomes in AECOPD. One case series with evidence of blood transfusion with Hb target more than 12 gm/dl facilitating successful weaning from the ventilator [20,21]. Thus, suggesting a role of Hb in AECOPD without clearly identified mechanisms. These findings led us to dig more into the relationship between anemia and AECOPD in terms of resource utilization, healthcare-related cost, mortality, and LOS from the NIS data to include the largest patient population.

AECOPD is the major cause of hospital admission for COPD patients. Patients with COPD exacerbations are usually treated with bronchodilators, antibiotics, and corticosteroids but in non-responders, a higher level of ventilator support is required by invasive or non-invasive mechanical ventilation [22]. We found the use of non-invasive mechanical ventilation in AECOPD is rising every year since the last decade compared to invasive ventilation (the trend for the years 2008 to 2014 in Figures 3, 4). This finding is supported in other studies as well in terms of better outcomes, lesser LOS, and reduced cost of hospitalization with NIPPV compared to IMV [23]. NIPPV not only helps in gas exchange, reducing carbon dioxide levels in the body, but it also facilitates respiratory muscle rest and reduces the workload of respiratory muscles. This enhances the benefits of other standard treatments and helps respiratory muscles to recover [23]. Furthermore, we found that anemia patients required more invasive as well as non-invasive ventilation support compared to non-anemia patients. Anemia is related to the work of breathing and minute ventilation [24]; the function of gas exchange and exercise capacity in COPD patients [25]. These associations may explain the higher need for supportive ventilation in anemia patients with AECOPD. There may be many complex pathophysiological mechanisms associated with this finding that needs to be explored in further basic science research studies. NIPPV can be implemented outside the ICU setting; thus, it helps to reduce the consumption of total ICU beds as well. However, the proper training of the staff and monitoring of these patients for treatment failure are very important to identify the need for IMV if require.

We found that the LOS is longer in anemia patients. The need for blood transfusion is also very high with an OR of 16.9 in anemic patients which may be related to a higher rate of adverse outcomes by blood transfusion-related complications at a certain level. Overall, it shows higher resource utilization by this subgroup of AECOPD patients, and this increasing demand in case management is reflected by the rising cost of hospitalization. The cost of hospitalization is nearly 40% higher in anemia patients compared to non-anemic patients with AECOPD. The cost of AECOPD in 2010 was reported as 14 billion dollars in the United States only, signifying the impact on the healthcare system [26], and it is increasing every year. Anemia is one of the common comorbidities, which can be easily identified, and measures to control anemia, as well as other similar comorbidities, would certainly affect the quality of life, AECOPD hospital adverse events, and cost of healthcare.

One important finding we came across in the analysis is decreasing rate of overall mortality in AECOPD patients. Interestingly, anemia patients had a higher mortality rate compared to non-anemia patients (1.2% vs 2%). The higher mortality could be related to the higher need for ventilation, blood transfusion, and complications related to that anemia patients.

This study has some limitations as well, despite high power due to a large number of patients from a national sample. The retrospective nature of the study has inherent limitations that can be present here in our study including coding errors, omissions, and unmeasured confounding factors. Anemia was identified from the ICD-9 codes, but it is unlikely that every anemia patient based on a Hb value less than 12 gm/dl would be coded in the database, especially in presence of associated multiple comorbidities. Patients with post-hemorrhagic anemia were not included in our study to strengthen study outcomes. Due to these facts, it is unlikely that the prevalence of anemia in AECOPD patients is overestimated in this study but it is likely that it is underestimated. It is not possible to determine whether the same individual was admitted multiple times due to a lack of identifiers in this database and this limits the identification of possible doubling of the same individual with AECOPD.

## Conclusions

The findings of our study indicate that anemia has a significant role to play in AECOPD and it is a commonly associated comorbidity. The presence of anemia in AECOPD patients is clearly associated with increased utilization of invasive and non-invasive modes of ventilation, longer LOS, and a significantly higher financial burden on the healthcare system. Anemia is associated with higher mortality compared to non-anemic patients, but it is satisfactory that the overall mortality rate is going down. We find that overall AECOPD cases are decreasing steadily from years 2008 to 2014 with consistently increasing use of NIPPV compared to IMV in hospitalized patients. Anemia is a commonly identifiable and treatable condition that can be focused on along with other comorbidities to reduce the adverse outcomes in AECOPD. Therefore, we propose a need for prospective studies on this topic and the development of effective interventions and options to early identify and manage the anemia prevalent with COPD.
